# A pilot randomized controlled trial of transcranial direct current stimulation adjunct to moderate-intensity aerobic exercise in hypertensive individuals

**DOI:** 10.3389/fnrgo.2024.1236486

**Published:** 2024-04-10

**Authors:** Edson Silva-Filho, Marom Bikson, Nigel Gebodh, Niranjan Khadka, Amilton da Cruz Santos, Rodrigo Pegado, Maria do Socorro Brasileiro-Santos

**Affiliations:** ^1^Associated Postgraduate Program in Physical Education, Federal University of Paraíba, João Pessoa, Paraíba, Brazil; ^2^Postgraduate Program in Physiotherapy and Postgraduate Program in Health Science, Federal University of Rio Grande do Norte, Santa Cruz, Brazil; ^3^Department of Biomedical Engineering, The City College of The City University of New York, New York, NY, United States

**Keywords:** blood pressure, heart rate, transcranial direct current stimulation, exercise, hypertension, sleep

## Abstract

**Background:**

Hypertension is a global issue that is projected to worsen with increasingly obese populations. The central nervous system including the parts of the cortex plays a key role in hemodynamic stability and homeostatic control of blood pressure (BP), making them critical components in understanding and investigating the neural control of BP. This study investigated the effects of anodal transcranial direct current stimulation (tDCS) associated with aerobic physical exercise on BP and heart rate variability in hypertensive patients.

**Methods:**

Twenty hypertensive patients were randomized into two groups: active tDCS associated with aerobic exercise or sham tDCS associated with aerobic exercise. BP and heart rate variability were analyzed before (baseline) and after twelve non-consecutive sessions. After each tDCS session (2 mA for 20 min), moderate-intensity aerobic exercise was carried out on a treadmill for 40 min.

**Results:**

A total of 20 patients were enrolled (53.9 ± 10.6 years, 30.1 ± 3.7 Kg/m^2^). There were no significant interactions between time and groups on diastolic BP during wake, sleep, over 24 and 3 h after the last intervention. Heart rate variability variables showed no significant difference for time, groups and interaction analysis, except for HF (ms^2^) between groups (*p* < 0.05).

**Conclusion:**

Anodal tDCS over the temporal cortex associated with aerobic exercise did not induce improvements in BP and heart rate variability.

**Clinical trial registration:**

https://ensaiosclinicos.gov.br/rg/RBR-56jg3n/1, identifier: RBR-56jg3n.

## Introduction

Hypertension is a global, chronic condition that affects ~25% of adults between 20 and 79 years (Zhou et al., 2021a). The prevalence of hypertension is divergent between developed and undeveloped countries. Approximately 350 million individuals experience hypertension in developed countries, and ~35% more cases are seen in undeveloped countries (Zhou et al., 2021a,b). Patients with hypertension also present with several comorbidities, including increased risk of stroke, neurovascular dysfunction, and cognitive impairments (Olsen et al., 2016; Zhou et al., 2021b).

Hypertension is typically treated with a regiment consisting of pharmacological and non-pharmacological interventions (Pescatello et al., 2019; Flack and Adekola, 2020). Aerobic exercise can reduce inflammatory processes and modulate heart rate variability (Masson et al., 2015; Besnier et al., 2017). Cardiovascular functioning can be managed through sympathovagal balance generated by physical exercise (Besnier et al., 2017), and the engagement of the sympathetic nervous system serves as a mechanism of action for alleviating hypertension (Hirooka, 2020). Several studies have investigated different means of treating hypertension with exercise by modulating the sympathetic nervous system. However, these studies have yet to present concrete recommendations for clinical use and application (Almeida et al., 2021). Interventions focused on cortical control of the autonomic nervous system have become crucial for improved understanding and management of blood pressure. Studies suggest that stimulation or inhibition of specific brain areas can decrease blood pressure and change autonomic responses in hypertensive patients (Rodrigues et al., 2021; Silva-Filho et al., 2021). Neuromodulation paired with aerobic exercise may acutely dampen hypertensive effects (Heinz et al., 2020; Silva-Filho et al., 2021).

Transcranial direct current stimulation (tDCS) is a safe and low-cost neuromodulation method that is used to interact with the central and autonomic nervous systems (Okano et al., 2015; Morya et al., 2019). tDCS is suggested to improve neuroplasticity in areas related to blood pressure control, such as the insular cortex, thalamus, and the medial prefrontal cortex (Okano et al., 2015; Farinatti et al., 2019; Silva-Filho et al., 2021). The insular cortex can be targeted with tDCS via electrodes placed over the temporal cortex (T3 area according to electroencephalogram 10/20). Okano et al. (2015) showed that anodal tDCS over the temporal cortex can modulate the autonomic nervous system in athletes by increasing parasympathetic activity. The temporal cortex is involved in the control of autonomic cardiac function and perceived exertion (Okano et al., 2015), making it an ideal target in investigating the association between tDCS and aerobic exercise to improve blood pressure in hypertensive individuals. It is important to highlight that tDCS was able to decrease sympathetic activity and increase the vagal modulation of hypertensive individuals, leading to a fall in peripheral vascular resistance and an adjustment in blood pressure and heart rate variability (Heinz et al., 2020; Rodrigues et al., 2021).

Although several successful pharmacological approaches have been adopted to control blood pressure, some critical intervention aspects must be considered when applying new types of treatment. Low-cost, safe, and effective non-pharmacological treatments targeting the central nervous system to control blood pressure are necessary to circumvent the use and dependence on pharmacological interventions. We hypothesize that the application of tDCS associated with aerobic exercise can decrease hypertensive metrics in patients affected by hypertension. Moreover, tDCS can be considered a non-pharmaceutical associated strategy with pharmacological approaches to manage hypertension. As the association between the targeted cortical area and effects on blood pressure are not well-understood, as well as the number of tDCS and aerobic exercise sessions required to reach a clinically meaningful effect on blood pressure, this study aimed to investigate the effects of anodal tDCS over the temporal cortex associated with moderate-intensity aerobic exercise on blood pressure and heart rate variability of hypertensive individuals.

## Methods

### Study design

We conducted a doubled-armed, parallel, triple-blinded pilot clinical trial. All procedures followed the Consolidated Standards of Reporting Trials (CONSORT) recommendations (Schulz et al., 2010), the declaration of Goodyear et al. (2007), and resolution No. 466/12 of the National Health Council. The local Ethics Committee approved this study (under ID: 3.101.577). After that, we registered it on the Brazilian Clinical Trials platform (RBR-56jg3n). All participants signed the informed consent prior to trial enrolment.

### Participants

The study was conducted at the Laboratory of Physical Training Studies Applied to Health at the Federal University of Paraíba between December 2019 and April 2020. Participants were recruited through advertisements in primary health care units and included according to the following criteria: (1) either sex; (2) over the age of 18; (3) had an arterial hypertension diagnosis according to ambulatory blood pressure monitoring (24 h measurement; systolic blood pressure> 120 <160 mmHg and/or diastolic blood pressure> 80 <100 mmHg). We excluded individuals who (1) presented with a history of hematological or neurological diseases; (2) were taking beta-blocking medications; (3) had any presence of peripheral vascular diseases; (4) previously had a stroke; (5) had cognitive damage by any cause; (6) had dissecting aneurysm; (7) had epileptic issues; (8) had seizures; (9) had physical limitations that interfered with our exercise protocol; (10) had metallic implants in the head; (11) were pregnant or lactating; (12) had alterations in their drug therapy for <2 months were excluded; (13) consumed more than 30 g of alcohol or 200 mg of caffeine per day; (14) began using any hormone replacement therapy; (15) modified the class or dosage of antihypertensive drugs.

### Randomization and blinding

**Figure 1:**
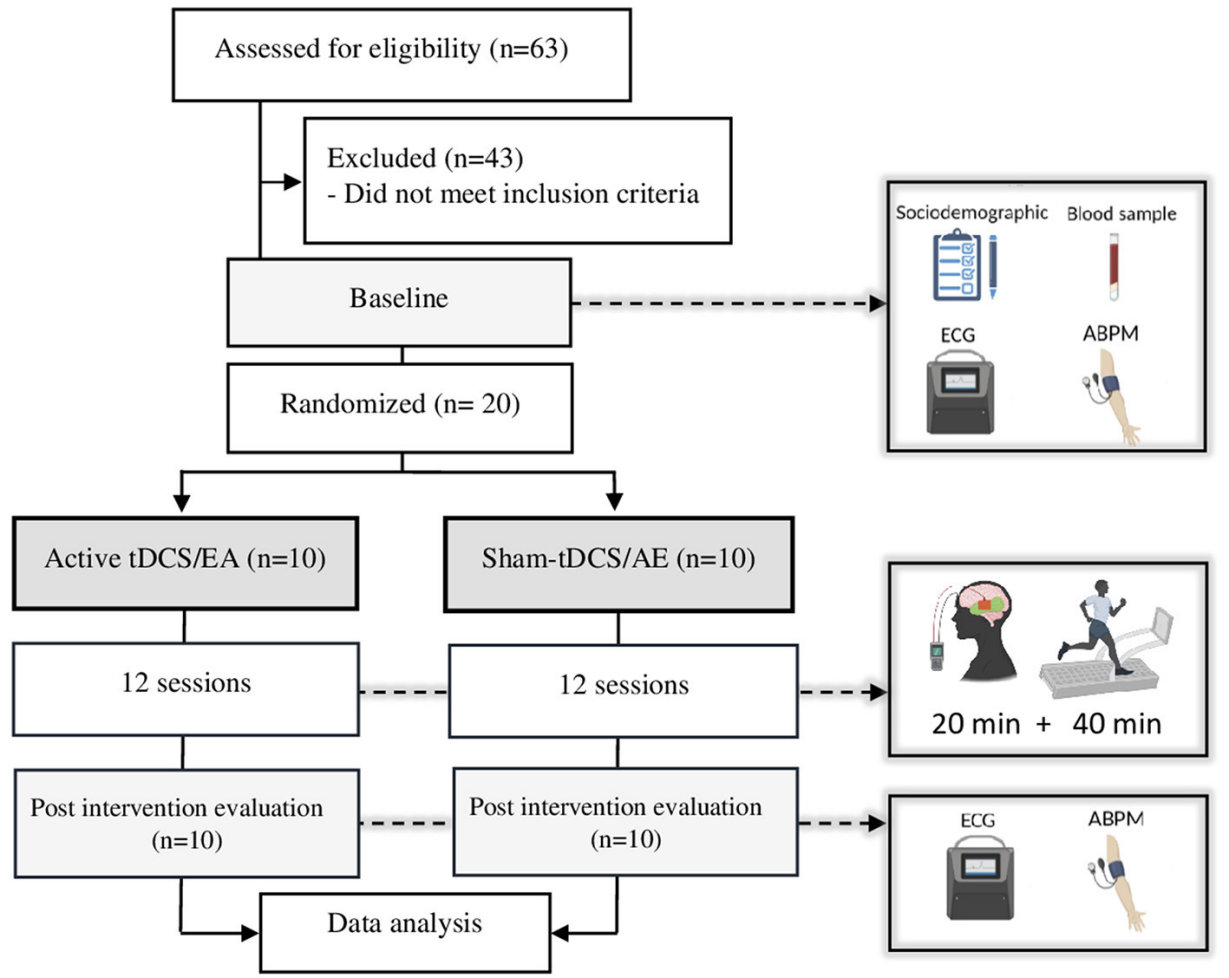
Study flowchart. AE, aerobic exercise; tDCS, transcranial direct current stimulation; ECG, electrocardiogram; ABPM, ambulatory blood pressure monitoring.

Initially, researchers considered sixty-three participants potentially eligible to participate in the study. After Ambulatory blood pressure monitoring measurement, twenty participants were allocated to the active-tDCS (*n* = 10) or sham-tDCS (*n* = 10) groups through a numerical sequence created by the www.random.org platform (Figure 1). An individual not otherwise involved in the study performed the randomization process. The allocation concealment was carried out through opaque envelopes and access to this content was allowed only by the researcher who was following the participants during the trial.

Blinding procedures ensured participants remained unaware of the stimulation type (active or sham tDCS). Sham tDCS consisted of 30 s of ramping stimulation up (to 2 mA) and down, similar to the active stimulation condition (Woods et al., 2016). For baseline and post-intervention, a blinded physiotherapist to the type of tDCS received by each participant assessed the outcomes. A third blinded researcher who did not participate in the study protocol conducted the statistical analysis.

### Intervention

Three times per week (Mon/Wed/Fri or Tue/Thu/Sat), both groups performed twelve non-consecutive sessions involving tDCS coupled with aerobic exercise. Each session initiated with 20 min of either active or sham tDCS, followed by completing 40 min of moderate-intensity aerobic exercise (Figure 1). Monitoring of heart rate variability and blood pressure occurred before and after the final session.

For tDCS procedures, participants seated in a comfortable chair with back and arm support. They received tDCS with the anode electrode positioned over the left temporal lobe (T3) and the cathode electrode over the contralateral supraorbital area (FP2), following international standards for the 10–20 EEG system. A sponge electrode, measuring 35 cm^2^, was immersed in ~12 ml of saline solution and connected to a tDCS device controlled by a digital multimeter (DT832, Wei Hua Electronic Co., Ltd, China) with a standard error of ±1.5%. The device delivered a constant 2 mA direct current for 20 min. The sham-tDCS group followed the same procedures, except for the 30-s current ramp up and down at the beginning of the intervention (Woods et al., 2016).

Immediately following each tDCS session, participants engaged in 40 min of non-inclined aerobic exercise on a treadmill (Athletic 430ee Advanced, USA). The exercise session protocol involved a 5-min warm-up at 57–63% of maximum/peak heart rate, followed by an intensity increase to 64–76% of maximum/peak heart rate (moderate intensity) for 30 min. The session concluded with a 5-min cool down at 57–63% of maximum/peak heart rate. The entire regimen adheres to the guidelines set by the American College of Sports Medicine (category A of evidence) for moderate-intensity aerobic exercise in hypertensive individuals (Garber et al., 2011).

### Clinical outcomes

At baseline, we measured sociodemographic data, collected blood samples, assessed blood pressure, analyzed heart rate variability, and evaluated aerobic capacity. Sociodemographic data include age, weight, height, abdominal circumference, sex, tabagism, educational level, marital status, comorbidities (diabetes), and the use of anti-hypertensive drugs. The values of weight and height, collected by an electronic scale coupled with a Welmy™ model W200 stadiometer, determined participants' body mass index.

A trained phlebotomist drew blood samples for biochemical tests. Biochemical analyses of glucose, blood count, total cholesterol and fractions, and triglycerides followed the International Council for Standardization in Haematology et al. (2014).

To find the heart rate/target training zone for each participant, an experienced cardiologist monitored the participants' aerobic capacity while they ran/walked on a treadmill. The speed of the treadmill increased every 2–3 min, totaling 8–12 min. The cardiologist immediately interrupted the test if the participant experienced tiredness, exhaustion, symptoms indicative of cardiovascular abnormalities, changes compatible with ischemia, significant changes in heart rhythm, or reached maximum heart rate.

Ambulatory blood pressure monitoring (Dyna-MAPA—CARDIOS™, Brazil) measured blood pressure for 24 h (including awake and sleep periods), in accordance with the recommendations from the European Society of Hypertension Practice Guidelines for home blood pressure monitoring (Parati et al., 2010). A cuff placed on the non-dominant arm, and a belt attached to the ambulatory blood pressure monitoring device equipped the participants. Participants recorded (write down in a diary) all the events that occurred throughout the day, such as physiological or psychological stresses, meal times, sleep, and wake. It is important to highlight that we measured and evaluated systolic and diastolic blood pressure during wake and sleep times, over 24 and 3 h, and after the last intervention.

For heart rate variability, the Holter electrocardiogram (CARDIOS™, Brazil) in the bipolar DII derivation (3 electrodes) recorded the electrocardiogram for 15 min in a supine position. Only the last 5 min of the acquired data were used for analysis. The variables square root of the mean squared difference of successive RR intervals (rMSSD), the standard deviation of RR intervals (SDNN), and triangular interpolation of NN interval histogram (TINN) were acquired as time-domain cardiac measures. The very-low-frequency (VLF; 0.0033–0.04 Hz), low frequency (LF ms^2^ and nu; 0.03–0.15 Hz), high frequency (HF ms^2^ and nu; 0.15–0.40 Hz), and the autonomic balance from the ratio between HF and LF were acquired as frequency cardiac measures.

### Statistical methods

Jamovi (3rd generation; version 2.3.28) analyzed the data, and GraphPad Prism (version 9.3.1) generated the data visualization. The student's *t*-test and the Chi-square test analyzed the baseline demographic characteristics and clinical scores between groups. The generalized mixed model analyzed the data (systolic and diastolic blood pressure during wake, sleep, 24 h, and after 3 h; rMSSD, SDNN, TINN, VLF, LF, HF and LF/HF) before and after the interventions. The dependent variables presented a non-symmetrical distribution, so the Gamma distribution with link function identity represented them. The independent factors encompassed time, group, and the interaction between the groups. The general mixed model with a random effect added to the constant of the model identified the individual variability. The mean difference, mean, standard deviation, standard error, and *p*-value represented the descriptive statistics for all baseline and post-intervention variables. For all analyses, we inserted body mass index as a covariant, and the significance level adopted was *p* < 5%.

## Results

A total of sixty-three participants were screened for eligibility. We excluded forty-three participants for not meeting the inclusion criteria. Twenty patients were randomized to each group (active or sham) and all of them completed all experimental procedures. All participants tolerated tDCS and aerobic exercise well. Socio-demographic and clinical data of both groups are summarized in Table 1. There were no differences between groups for all variables, except body mass index (*p* < 0.05).

**Table 1:** Socio-demographic and clinical data.

**Outcomes**	**Active tDCS + PE (*n* = 10)**	**Sham tDCS + PE (*n* = 10)**
Age	56.8 ± 7.5	51.1 ± 12.8
BMI	29.4 ± 1.8	30.8 ± 4.9
Abdominal circumference (cm)	99.3 ± 7.1	102.9 ± 12.6
**Systolic blood pressure**		
Vigil	134 ± 10.2	131 ± 6.8
Sleep	124 ± 11.6	116 ± 11.8
24 h	132 ± 10.5	128 ± 7.2
After 3h	132 ± 9.5	137 ± 11.4
**Diastolic blood pressure**		
Vigil	81.8 ± 9.07	76.7 ± 10.3
Sleep	71.9 ± 11.9	65.4 ± 11.1
24 h	79.6 ± 9.3	74.5 ± 9.9
After 3h	81.3 ± 10.1	81 ± 11.5
**Sex**		
Male	30%	40%
Female	70%	60%
Smoke (no %)	80%	90%
**Schooling**		
Elementary school	30%	50%
High school	30%	30%
Undergraduate	40%	20%
**Civil status**		
Married	70%	60%
Single	0%	30%
Divorced	10%	10%
Widow	20%	0%
Comorbidities (diabetes)	30%	20%
**Antihypertensive drugs**		
No drugs	40%	30%
1 drug	10%	30%
2–3 drugs	40%	40%
≥4 drugs	10%	0%
**Biochemical tests**		
Red-blood (μ/L)	4.67 ± 0.44	4.49 ± 0.57
Hemoglobin (g/dL)	13.9 ± 0.91	13.1 ± 1.8
Hematocrit (%)	42.1 ± 3.1	40.6 ± 5.7
Glucose (mg/dL)	103.4 ± 35.3	108.2 ± 59.4
Monocytes (μ/L)	317.8 ± 190.7	212.1 ± 95.1
Leukocytes (μ/L)	6,998.5 ± 1,716.1	6,830.0 ± 936.2
Lymphocytes (μ/L)	2,735.8 ± 910.1	2,500.4 ± 827.7
Platelets (μ/L)	243.1 ± 25.9	230.5 ± 54.2
Total cholesterol (mg/dL)	203.1 ± 59.6	178.0 ± 32.4
HDL-cholesterol (mg/dL)	51.7 ± 15.4	54.8 ± 18.3
LDL-cholesterol (mg/dL)	123.8 ± 51.8	105.2 ± 29.9
Triglycerides (mg/dL)	175.5 ± 113.4	164.6 ± 62.7

PE, physical exercise; data presented in mean and standard deviation; %, percentage; cm, centimeters; μ/L, units per liter; mg/dL, milligrams per deciliter; BMI, body mass index.

**Figure 2:**
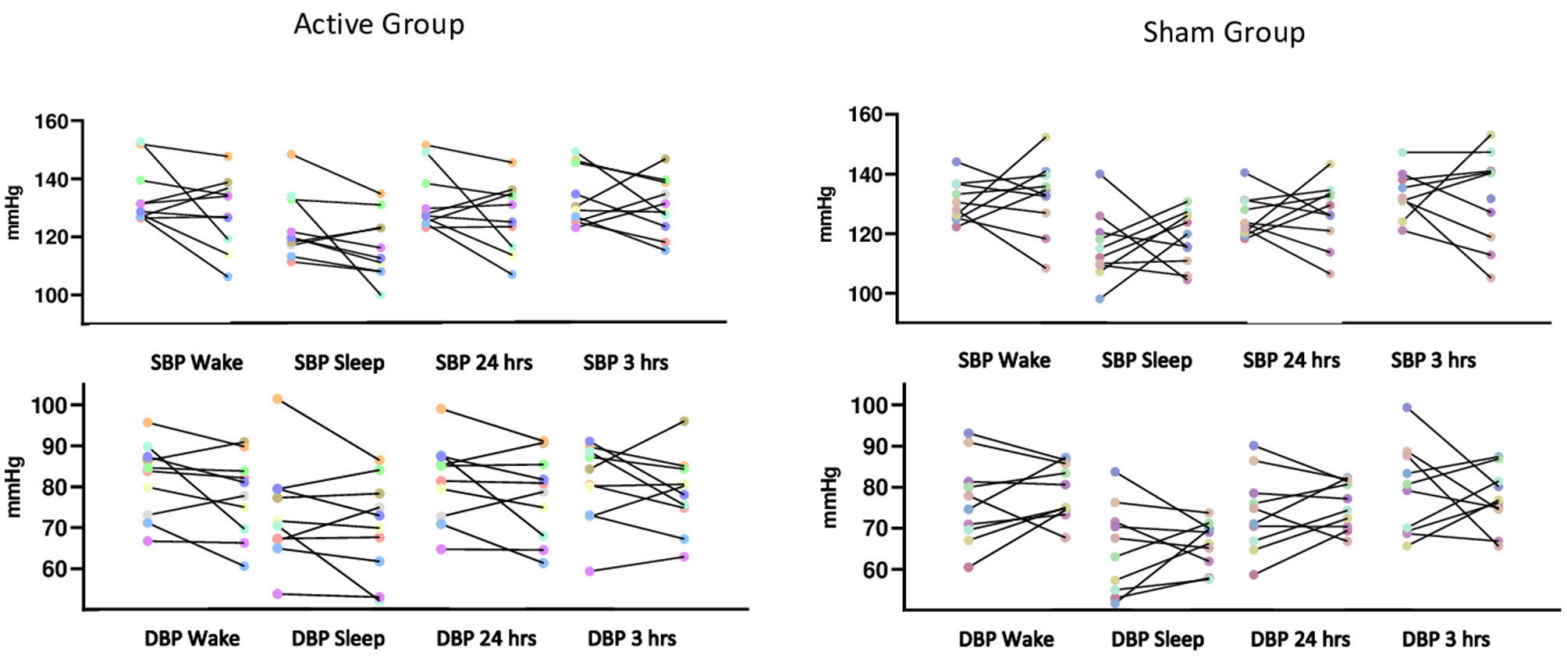
Systolic and diastolic blood pressure from each individual assessed before and after the interventions during awake and sleep times, during 24 and 3 h after the last intervention.

The generalized mixed model showed no statistically significant interaction between groups on blood pressure after interventions during wake, sleep, over 24 and 3 h after the last intervention (Table 2). It is important to note that systolic and diastolic blood pressure individual responses, considering baseline and post-interventions, showed high variability (Figure 2). Heart rate variability variables showed no significant difference for time, groups and interaction analysis, except for HF (ms^2^) between groups (*p* < 0.05; Table 3).

**Table 2:** Between groups analysis of systolic and diastolic blood pressure during wake and sleep time, 24 and 3 h after interventions.

**Blood pressure**	**Mean difference (CI)**	**Std. error**	***p*-value**
SBP wake	0.5 (−9.35; 10.46)	5.0	0.91
DBP wake	−1.7 (−11.49; 8.09)	4.9	0.73
SBP sleep	−1.9 (−11.5; 7.6)	4.8	0.69
DBP sleep	−4.1 (−15.1; 6.8)	5.6	0.45
SBP 24 h	−0.1 (−9.7; 9.4)	4.8	0.98
DBP 24 h	−2.0 (−11.7; 7.5)	4.9	0.67
SBP after 3 h	5.0 (−5.9; 16.0)	5.6	0.36
DBP after 3 h	0.5 (−8.9; 10.0)	4.8	0.91

SBP, systolic blood pressure; DBP, diastolic blood pressure; confidence interval: 95%. SE, standard error. Mean difference between sham and active group.

**Table 3:** Between groups analysis of heart rate variability variables after interventions.

**Outcomes**	**Mean difference (CI)**	**Std. error**	***p*-value**
VLF (ms^2^)	−13.3 (−152.1; 125.3)	70.7	0.85
LF (ms^2^)	−42.7 (−144.7; 59.2)	52.0	0.41
LF (nu)	−11.2 (−30.3; 7.7)	9.7	0.24
HF (ms^2^)	309.1 (116.5; 501.6)	98.2	<0.05
HF (nu)	12.2 (−7.0; 31.4)	9.8	0.21
LF/HF (%)	−0.4 (−1.2; 0.2)	0.3	0.18
rMSSD (ms)	4.6 (−10.0; 19.4)	7.5	0.53
SDNN (ms)	2.5 (−9.7; 14.7)	6.2	0.68
TINN (ms)	3.1 (−34.9; 41.2)	19.4	0.87

ms^2^, meters per seconds squared; ms, meters per second; nu, normal units; %, percentage; VLF, very low frequency; LF, low-frequency band; HF, high- frequency band; rMSSD, square root of the mean squared difference of successive RR intervals; SDNN, standard deviation of RR intervals; TINN, triangular interpolation of NN interval histogram. CI: 95%, SE, standard error. Mean difference between sham and active group.

## Discussion

In this study, we introduced a pioneering trial combining twelve sessions of tDCS with aerobic exercise to improve the blood pressure and heart rate variability in hypertensive individuals. Following the intervention, our analysis revealed no significant results in blood pressure and heart rate variability across the groups. Despite the initial appearance of these outcomes as discouraging, they hold significant importance in advancing research within this domain. As aerobic exercise has been considered a gold standard non-pharmacological treatment to decrease blood pressure in hypertensive individuals (Besnier et al., 2017; Pescatello et al., 2019), we hypothesize that the ceiling effect generated by 40-min physical exercise made tDCS hard to potentiate the results (Lopes et al., 2021).

The use of tDCS and aerobic exercise for modulating autonomic nervous activity was previously reported for young men (Farinatti et al., 2019), post-stroke (Heinz et al., 2020), athletes (Montenegro et al., 2011), and hypertensive participants (Rodrigues et al., 2021), however these studies consisted of single session interventions. Consequently, they only evaluated the immediate short-term effect of tDCS and aerobic exercise (Montenegro et al., 2011; Farinatti et al., 2019; Heinz et al., 2020; Rodrigues et al., 2021). Non-invasive brain stimulation strategies, such as repetitive transcranial magnetic stimulation and tDCS have been tested to modulate the brain-heart pathway in cardiovascular disease (Makovac et al., 2017). It has been suggested that non-invasive brain stimulation is an effective tool for controlling blood pressure and heart rate variability (Makovac et al., 2017). The stimulation dose, including cortical target, current intensity, and the number of sessions play a crucial role in cardiovascular responses (Makovac et al., 2017).

A few trials tested the vagal stimulation effects on outcomes such as sleep (Jackowska et al., 2022), chronic pain (Muthulingam et al., 2021), and inflammatory response after surgery (Salama et al., 2020). Also, there is an ongoing trial, phase two, using transcutaneous vagal stimulation on adult patients with uncontrolled high blood pressure (Gupta, 2022). Although a few clinical trials analyzed the efficacy of vagal stimulation, the mechanism behind it is poorly understood. It is suggested that the effects of vagal stimulation in the heart involve the preganglionic nerve stimuli to activate synapses on parasympathetic ganglia in the epicardium and cardiac septum. The activation of parasympathetic ganglionic neurons is essential due to their postganglionic axon projections to the cardiac atrium, ventriculus, and all the structures in these regions, such as sinoatrial and atrioventricular nodes (Capilupi et al., 2020). So, theoretically, parasympathetic regulation through vagal and correlated areas of stimuli holds substantial significance in blood pressure control.

Different central and peripheral mechanisms have been described as important components to control blood pressure (Cogiamanian et al., 2010). One of the mechanisms involves heart rate variability analysis, which represents the autonomic function. Considering the up-down regulation, sympathetic and parasympathetic activity modulation by the insular cortex through projections over the solitary tract nucleus, ventrolateral rostral medulla, and periventricular/periaqueductal gray areas is a potential pathway involving in the cardiovascular adjustment (Cogiamanian et al., 2010). Based on these mechanisms, it is expected that non-invasive brain stimulation techniques, such as tDCS, interfere with cardiac parameters and decrease the blood pressure of hypertensive individuals.

A significant decrease in the high frequency (ms^2^) for the active tDCS associated with aerobic exercise was detected. However, there was a difference between the groups before the intervention. Previous studies with one session of aerobic exercise preceded by tDCS, applied over medial prefrontal cortex, induced postexercise hypotension in normotensive men (Farinatti et al., 2019). The authors found a significant decrease in systolic blood pressure and mean arterial pressure during 60-min of post-exercise recovery (Farinatti et al., 2019). Another study with hypertensive patients showed a positive acute adjustment in autonomic cardiac control and reduction in 24 h of blood pressure measurements after a single session of tDCS (C3/FP2 montage) (Rodrigues et al., 2021).

It is important to mention that the association between tDCS and aerobic exercise showed effects on controlling blood pressure and heart rate variability in young men (Farinatti et al., 2019). Nonetheless, some authors demonstrated that tDCS was not able to potentiate the aerobic exercise effects over systolic blood pressure and heart rate variability of hemiparetic patients (Heinz et al., 2020). These studies showed diverged results, possibly due to differing dosing parameters and stimulation target locations, the medial prefrontal (Farinatti et al., 2019), and the left temporal cortex (Heinz et al., 2020). Thus far, there is no standard stimulation target location to effectively engage the central nervous system to alter blood pressure and heart rate variability response.

Okano et al. (2015) previously showed the effect of tDCS over the T3 on the autonomic nervous system, rating of perceived exertion, and performance during a maximal dynamic exercise in trained cyclists. The authors suggest that brain stimulation over the temporal cortex during exercise modulates autonomic nervous system activity, sensory perception of effort, and exercise performance (Okano et al., 2015). They also demonstrated that stimulation targeting the left insular cortex increased parasympathetic modulation (Okano et al., 2015). These cortical stimulation targets are associated with better control of heart rate variability; however, little is known about the stimulation effect of the temporal and insular cortex on blood pressure (Okano et al., 2015; Silva-Filho et al., 2021). In a study with athletes and non-athletes, anodal tDCS applied over the temporal cortex significantly increased parasympathetic activity and decreased sympathetic activity in endurance-trained athletes, but no changes were observed for non-athletes (Montenegro et al., 2011). Thus, some parameters, including the aerobic exercise intensity, individual aerobic capacity, the target of stimulation, and the number of sessions, could help explain the results.

Innovation in the research field is challenging. Although there is a gap in the literature and a theory that supports the use of tDCS in hypertensive people, the human body and the technique parameters are complex. This is the first trial that mirrors clinical practice using tDCS associated with one of the non-pharmacological gold standard treatments for people affected by hypertension. We believe that several parameters must be adjusted in an attempt to reach the best results. Therefore, considering these assumptions, we can learn from null outcomes to improve the upcoming trials.

Some limitations of the present study must be acknowledged. We recognize that our modest sample size could not differentiate all effects of tDCS associated with moderate aerobic exercise on blood pressure and heart rate variability, increasing the risk of bias due to individual variability. Also, for all outcomes the results are powerless, so we cannot perform inferences. Long-term effects of hypertension can affect blood pressure outcomes, making reduction in such measures more difficult for chronic sufferers. The patient's time of diagnosis and treatment were not explicitly acquired throughout our trial. The eligibility criteria, randomization, and analysis did not consider the antihypertensive drug intake. However, future trials should account for these factors. It is imperative to underscore the absence of published studies providing a definitive basis for determining the sample size due to the divergence in methodologies presented. This deficit in prior information poses challenges in the accurate calculation of the sample size.

## Conclusion

Aerobic exercise associated with anodal tDCS over the temporal cortex did not reduce blood pressure during wake, sleep, over 24 h, and 3 h after the last session. Also, no effects were found for heart rate variability between active and sham groups, except for high frequency. This study explored the targeting of tDCS associated with aerobic exercise for understanding the application of integrative therapies using non-invasive brain stimulation in hypertension.

## Data availability statement

The original contributions presented in the study are included in the article/supplementary material, further inquiries can be directed to the corresponding author.

## Ethics statement

The studies involving humans were approved by Instituto de Educação Superior da Paraíba—IESP. The studies were conducted in accordance with the local legislation and institutional requirements. The participants provided their written informed consent to participate in this study.

## Author contributions

ES-F, MS, and AC: conceptualization. ES-F and MS: methodology. ES-F: investigation. All authors: writing—original draft and writing—review and editing. RP and MS: supervision. All authors contributed to the article and approved the submitted version.

## Supplementary material

The datasets presented in this study can be found in the Zenodo online repository, see below.

Silva-Filho et al. (2021) effects of neuromodulation of the insular cortex associated with physical exercise on cardiovascular function in hypertensive [Zenodo] (https://doi.org/10.5281/zenodo.8014762).
